# Multidimensional Study on the Wear of High-Speed, High-Temperature, Heavy-Load Bearings

**DOI:** 10.3390/ma16072714

**Published:** 2023-03-29

**Authors:** Dongfeng Wang, Julong Yuan, Lai Hu, Binghai Lyu

**Affiliations:** 1 College of Mechanical Engineering, Zhejiang University of Technology, Hangzhou 310023, China; 2 Luoyang Bearing Research Institute Co., Ltd., Luoyang 471039, China; 3 School of Mechanical Engineering, Xi’an Jiaotong University, 28 Xianning Road, Xi’an 710049, China; 4 State Key Laboratory for Manufacturing Systems Engineering, Xi’an Jiaotong University, Xi’an 710054, China

**Keywords:** high-performance bearings, experiment, multidimensional analysis, wear, performance

## Abstract

The friction and wear performance of high-performance bearings directly affects the accuracy and maneuverability of weapons and equipment. In this study, high-speed, high-temperature, and heavy-load durability experiments of weapon bearings were carried out, and their wear properties (i.e., surface wear, metamorphic layer, scanning electron microscopy/energy-dispersive spectroscopy (SEM/EDS), residual stress, and retained austenite) were analyzed in multiple dimensions. The results showed the following: (1) The experimental temperature of the serviced front-end bearing is always lower than that of the rear bearing. (2) The metamorphic layer of the serviced rear bearing (i.e., inner ring, outer ring, rolling body, and cage) > the metamorphic layer of the serviced front-end bearing > the metamorphic layer of the unserviced bearing. (3) The rolling body of the rear bearing at high experimental temperatures contains not only elemental O, but also elemental P and Sr. (4) In the EDS analysis of the rolling elements, with the migration from the “ball edge” to the “ball center”, the elemental C in the rolling elements of serviced or unserviced bearings decreases slowly, while the elemental Fe content increases slowly.

## 1. Introduction

The advanced nature and maneuverability of weapons and equipment is one of the symbols of national military strength, and the characteristics of high-performance bearings directly affect the advanced nature and maneuverability of weapons and equipment [1,2]. Because weapons and equipment are in continuous motion, high-performance bearings will rotate under various conditions, such as high temperature, high speed, heavy load, etc. In the research of bearings, many scholars have made several contributions.

Wang P et al. [3] designed an early-warning method for wear faults of rolling bearings based on empirical mode decomposition (EMD). After verification, the signal denoising effect of this method is good, the early-warning accuracy is always over 94%, and the average alarm time is close to 0.27 s. Alves D S et al. [4] analyzed the time response of rotating machinery with bearing wear in order to observe the vibration behavior of the bearing wear. Shi XiJiang et al. [5] put forward a combined numerical algorithm for judging the lubrication state of high-speed and heavy-duty angular-contact ball bearings of aero-engine spindles. The research showed that the bearings maintain the full oil film state under normal operation conditions, and short-term poor lubrication may occur under deceleration, start-up, or stop conditions. Zmarzy P [6] established a multidimensional mathematical model to evaluate the effects of selected factors on the vibration wear of 6304ZZ rolling bearings from three manufacturers. Shi X et al. [7] developed a numerical friction dynamics analysis program for predicting the dynamic performance, lubrication state, friction temperature, and surface stress of aviation ball bearings. The results showed that a large dry contact area, high friction temperature, high peak local stress value, and poor lubrication state of bearings at heavy loads and low speeds may lead to a large number of wear, scratch, and micro-pitting failures. Xi Zhang et al. [8] established a dynamic contact-wear model of ball bearings, composed of wear degree and position distribution. Yang Z et al. [9] studied the influence of the uncertainty of angular deviation on the wear of angular-contact ball bearings in a spindle system. The results showed that the uncertainty of the initial angular deviation leads to obvious dispersion of the wear depth of the ball bearings, but the degree of dispersion is related to the spindle speed, angular deviation, and bearing installation position.

In the field of fatigue life research, Shi X et al. [10] put forward a new method to calculate the relative fatigue life of bearings. The influence of the surface texture direction on subsurface stress and relative fatigue life was studied. Hiraki K et al. [11] carried out 93 N-387 N rolling-contact fatigue tests in the radial load range of 600 rpm. They found that the friction coefficient ranged from 0.013 to 0.032 in the whole test. Yang Z et al. [12] proposed an image-based evaluation method for cage stability. In order to accurately determine the motion trajectory of the bearing cage, a new experimental device was developed. The research results were concluded to provide reference for the structural optimization design and life prediction of high-precision ball bearings. Trivedi H K et al. [13] analyzed the friction film formed on the bearing surface and studied the lubricant additive. The research showed that the gas turbine lubricant prepared with tricresyl phosphate (TCP) should form a beneficial friction film to improve the fatigue life and performance of the bearings. Cheng Y et al. [14] proposed a new two-stage remaining useful life (RUL) prediction method based on depth learning, which uses fast search and find of density peaks clustering (FSFDPC) and a multidimensional deep neural network (MDDNN). The research showed that this method has good prediction performance under different working conditions. References [15,16,17,18] also predicted the wear, friction, oil film thickness, and lubrication of high-performance bearings through mathematical models. El Laithy et al. [19] carried out detailed mechanical research through scanning electron microscopy (SEM), electron backscatter diffraction (EBSD), and nanoindentation analysis, showing the evolution of ferrite grains (equiaxed grains and elongated grains) and the carbide structure in the network formed in the inner ring of the angular-contact ball bearings at different life stages. Abdullah et al. [20] analyzed and reported for the first time the bearing ball-to-ball point-contact loading conditions through comprehensive rolling-contact fatigue (RCF) data. The area fraction of the dark etched zone observed experimentally in rolling bearings has been evaluated according to the dark etching regions (DER%) and compared with the dislocation-assisted carbon diffusion model used for DER formation. Jouini et al. [21] studied the surface integrity of AISI 52,100 bearing rings after high-precision hard turning and grinding, along with its influence on fatigue life. The residual stresses measured in the RCF tests (after running-in and spalling) showed peak compression values at a depth of 140 μm underground. The fatigue life of rings machined by high-precision hard turning is four times that of those machined by grinding. Zhao et al. [22] compared the rolling-contact fatigue life of carburized and uncarburized 100 Cr6 alloy. The results showed that the life of RCF (L10) was increased from 1 × 107 cycles to more than 1 × 108 cycles by carburization. Zhang et al. [23] put forward a theoretical model to analyze the influence of friction on the rolling-contact fatigue life of angular-contact ball bearings. The relative RCF life of ball–raceway contact was calculated, and the accuracy of the calculation was confirmed by published research. Watanuki et al. [24] carried out fatigue tests on real bearings with various artificial defects on the outer-ring raceway. Through simulation, the gap fittings between the outer ring and the shell were determined, as well as the size of the initial defects, both of which will affect the fatigue threshold of the bearing. Lorenz et al. [25] studied the effects of various spatial hardness gradients (e.g., linear and nonlinear) on RCF life through a Mises-based plastic framework. The RCF results showed that the polynomial gradient with a degree greater than 1 was superior to the linear and polynomial gradients with a degree less than 1, and the difference between the gradient types was more obvious at shallower gradient depths. With the increase in the gradient depth and residual compressive stress, the RCF resistance was improved. Yang et al. [26] proposed an image-based cage stability evaluation method. In order to accurately determine the motion trajectory of the cage, a new experimental device was developed. The research results have a certain reference value for the structural design optimization and life prediction of high-precision ball bearings. Schwendemann et al. [26] discussed the most important methods for fault analysis of grinder bearings and introduced the prediction of remaining service life. Paiva et al. [27] analyzed the morphology of the ground surface and the microhardness below the machined surface. The results showed that the surfaces milled with semi-synthetic fluid usually had lower Ra values and microhardness changes. The model for the prediction of Ra showed a maximum error of 14% compared with the measured value. Sridharan et al. [28] reliably predicted that thermal damage in penetration-hardening bearing steels is independent of grinding variables. Through metallographic and residual stress analysis, various strengths of material transformation were accurately detected and verified. The results showed that material-specific models that are independent of the grinding process variables can be effectively used to predict thermal damage. Cui et al. [29] studied a new rolling-contact fatigue (RCF) life model of rolling bearings, which is helpful to promote the development of cyclic fatigue theory of rolling bearings. Duan et al. [30] established a coupling model—including a quasi-static model, a fatigue life model, and a mixed lubrication model—to study the effects of angular eccentricity on high-speed cylindrical roller bearings. Kong et al. [31] established a nonlinear dynamic model of a gear-bearing system. The dynamic model can calculate the internal load of the bearings and determine their fatigue life by using linear damage theory. Jouini et al. [21] investigated the surface integrity of AISI 52,100 bearing rings. The influence of high-precision hard turning and grinding on the fatigue life of the bearing rings was analyzed. The results showed that the fatigue life of ring specimens machined by high-precision hard turning is four times longer than that of those machined by grinding. Zhuang et al. [32] studied the fatigue life of raceways based on the ISO 281-2007 bearing life theory. The results showed that the friction on the contact surface has a certain influence on the stress and fatigue life.

**Figure 1:**
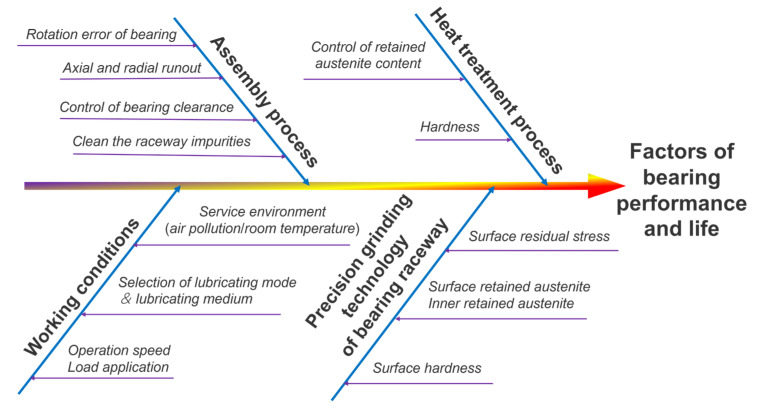
Ishikawa diagram of factors affecting bearings’ performance and life.

In addition to the above team’s research on bearing fatigue life, this study lists the factors that affect bearings’ performance and life, as shown in Figure 1.

As shown in Figure 1, the influence on high-performance bearings can be mainly divided into four important parts—namely, the heat treatment process, the precision grinding technology of the bearing raceway, the bearing assembly process, and the bearing service conditions. The key influencing factors are listed in each part. Because of the above research, a new research idea was proposed. High-speed, high-temperature, and heavy-load durability tests were conducted to study the performance of high-performance bearings during and after the experiments. The results were analyzed and compared from a multidimensional perspective, including the surface wear of the inner and outer rings, cages and balls, raceway metamorphic layer and element content, etc. Among them, microscopic changes in the surface quality of high-performance bearings were observed by SEM. The contents of the high-performance bearings before and after testing were compared and analyzed by EDS. Finally, combined with the experimental data and bearing life theory, the life influence of high-performance bearings was analyzed. The factors influencing the life of high-performance bearings under actual service conditions—such as the changes in the microscopic quality and element contents on the surface of the inner and outer rings and rolling bodies of the bearings—were comprehensively discussed. These findings provide data support and a research foundation for high-performance bearing manufacturers and scholars in related fields.

## 2. Multidimensional Experimental Analysis

**Figure 2:**
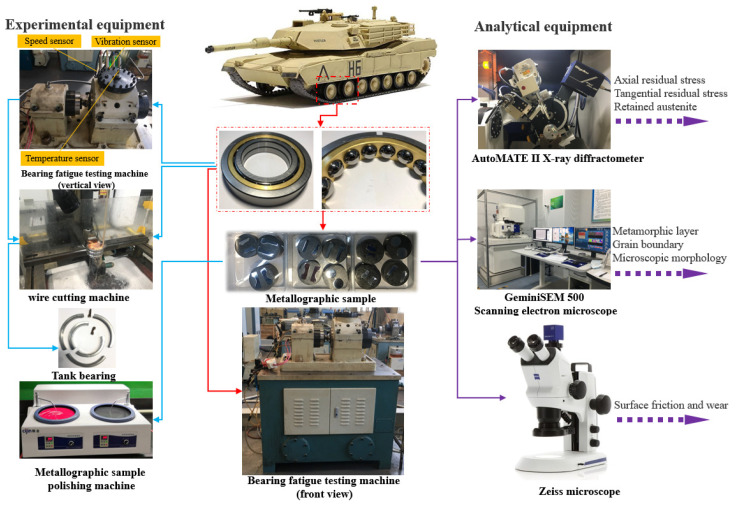
Experimental equipment and sequence of multidimensional analysis of high-performance bearings.

In this study, many advanced devices were used to carry out high-speed, high-temperature, and heavy-duty durability experiments on high-performance bearings. Furthermore, characterization equipment was used to inspect and observe the manufacture of the metallographic test pieces, as shown in Figure 2.

As shown in Figure 2, the T30-70Nf bearing testing machine (speed range: 0–120,000 r/min; temperature range: −300 °C~100 °C; load range: 0~30 T) was selected for high-speed, high-temperature, and heavy-load experiments. The TAT-400 wire-cutting machine tool was selected for the bearing cutting equipment (cutting efficiency: ≤220 mm/min; positioning accuracy: ≤0.01 mm; wire speed: 3–12 m/s; pulse resolution: 0.4 μm). The Zeiss Primotech microscope and Zeiss GeminiSEM 500 scanning electron microscope were selected for microscopic SEM and EDS. Residual stress and retained austenite were measured using a Rigaku AutoMATE II high-power micro-are X-ray diffractometer (2θ: 98°–168°; scanning accuracy: 0.1 μm; minimum step: 1/10,000; power: 3 KW). The model of high-performance bearing tested was QJ214 (bearing series: four-point angular-contact ball bearing; material: high-carbon chromium bearing steel GCr15; rated dynamic load: 106,000 N; rated static load: 197,000 N; the radial runout and axial runout of the bearing’s outer ring were 2 μm and 5 μm, respectively).

### 2.1. High-Speed Durability Experiment

**Figure 3:**
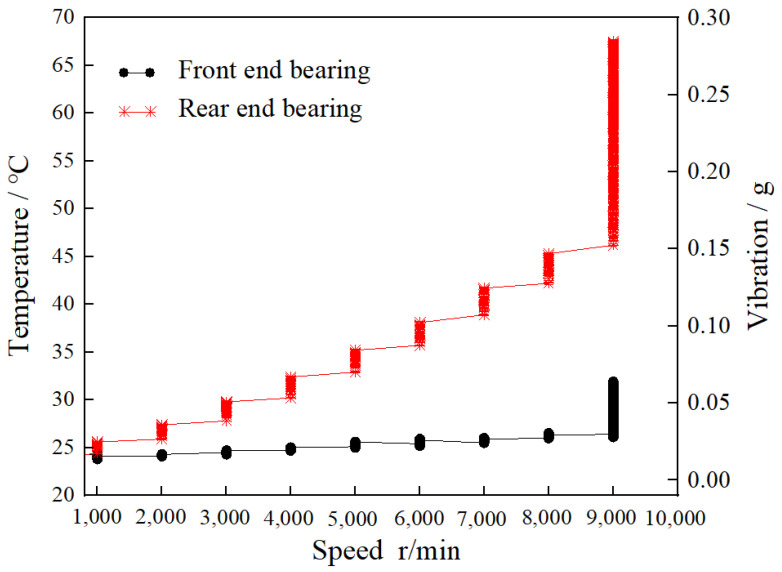
High-speed durability testing of high-performance bearings.

Since most bearings are used in pairs, a set of bearings (front-end and rear bearings) was selected and tested at high speed for 8 h continuously. In the experiment, the speed of 1000 r/min (fixed for 15 min) was continuously increased in the first two hours. After 2 h, the speed was 9000 r/min, where it was maintained for 6 h. Figure 3 shows the relationship between the bearing temperature and the vibration of high-performance bearings under high-speed operation.

According to the macroscopic analysis of Figure 3, the temperature and vibration of the front-end bearing did not change much in the first two hours, while the rear bearing increased in gradient. When the rotation speed reached 9000 r/min, the temperature and vibration of the front-end and rear bearings increased dramatically.

According to the microscopic analysis of Figure 3, the temperature of the front-end bearing was essentially stable at 24 °C, and the vibration was stable at 0.035 g in the first two hours. The temperature of the rear bearing started at 25 °C and increased by 2.5 °C when the rotation speed increased by 1000 r/min. In the process of increasing, the temperature increased sharply for a short time when it was maintained for 15 min, and the vibration increased with the increase in the rotation speed. When the rotation speed reached 9000 r/min, the temperature of the front-end and the rear bearings was 25.2 °C and 46.4 °C, respectively. The vibrations were 0.035 g and 0.15 g, respectively. With the speed (9000 r/min) maintained for 6 h, the temperature of the front-end and rear bearings ultimately reached 37.6 °C and 67.8 °C, respectively, and the vibrations ultimately reached 0.072 g and 0.282 g, respectively.

### 2.2. High-Temperature Durability Experiment

**Figure 4:**
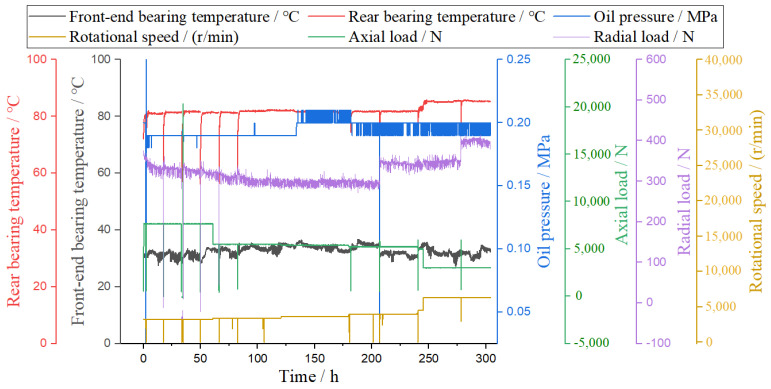
High-temperature durability testing of high-performance bearings.

High-performance bearings also bear internal friction and high temperature caused from the outside (such as heavy loads and temperature changes in different environments) [33]. In this study, we also carried out high-temperature durability testing of high-performance bearings in continuous operation for 300 h (the base temperature was 20 °C, and no external cooling was required during the test.). Comprehensive analysis was carried out, consisting of bearing temperature, oil pressure, axial load, radial load, and rotational speed experiments, as shown in Figure 4.

According to the macroscopic analysis of Figure 4, with the increase in speed and time, the temperature of the front-end bearing was always lower than that of the rear bearing. The oil supply pressure was relatively stable. The axial load and radial load fluctuated briefly.

According to the microscopic analysis of Figure 4, with the increase in the rotation speed (minimum rotation speed 3000 r/min, maximum rotation speed 6300 r/min) and time, the highest and lowest temperatures of the front-end bearings were 36.7 °C and 27.2 °C, respectively. The highest and lowest temperatures of the rear bearings were 85.8 °C and 52.4 °C, respectively. The highest and lowest oil supply pressures were 0.25 MPa and 0.18 MPa, respectively. The maximum and minimum axial loads were 7703 N and 382 N, respectively. The maximum and minimum radial loads were 414 N and 9 N, respectively.

### 2.3. Heavy-Load Durability Experiment

**Figure 5:**
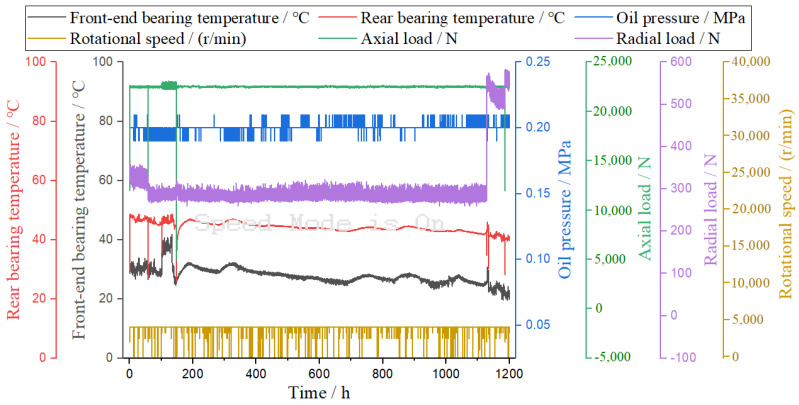
Heavy-load durability testing of high-performance bearings.

In addition to the high-speed and high-temperature experiments, the high-performance shafts were also subjected to heavy-load testing. In this study, the heavy-load durability testing of high-performance bearings was carried out for 1200 h continuously (the base temperature was 20 °C, and no external cooling was required during the test). Similarly, a comprehensive analysis was conducted, consisting of bearing temperature, oil pressure, axial load, radial load, and rotational speed experiments, as shown in Figure 5.

According to the macroscopic analysis of Figure 5, the speed was set to 4000 r/min, and with the increase in time, the temperature of the front bearing was generally lower than that of the rear bearing. The oil supply pressure was relatively stable. The axial load and radial load fluctuated briefly.

According to the microscopic analysis of Figure 5, at a constant speed (3000 r/min) and with the increase in time, the highest and lowest temperatures of the front-end bearings were 41.9 °C and 19.6 °C, respectively. The highest and lowest temperatures of the rear bearings were 48.8 °C and 26.1 °C, respectively. The highest and lowest oil supply pressures were 0.23 MPa and 0.06 MPa, respectively. The maximum and minimum axial loads were 23,076 N and 620 N, respectively, and the axial loads were typically maintained above 22,500 N. The maximum and minimum radial loads were 583 N and 259 N, respectively. The radial load increased sharply in the later period and lasted for 90 h, with the load ranging from 500 N to 593 N.

## 3. Comparative Analysis of the Surface Integrity of High-Performance Bearings

In this study, the high-speed, high-temperature, and heavy-load durability of high-performance bearings were tested and analyzed, as described in the previous section. In this section, after the durability experiments, we compare and analyze the serviced and unserviced bearing conditions, including the surface wear, metamorphic layer, and surface element contents of the inner and outer rings, cages, and rolling bodies of the bearings. Among them, the bearings that were tested are called serviced bearings. The bearings that were been tested are called unserviced bearings.

### 3.1. Surface Wear

**Figure 6:**
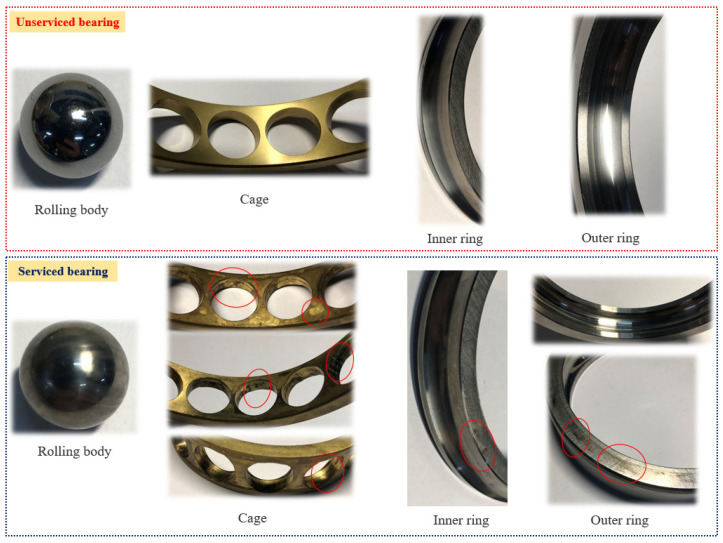
Naked-eye comparison of the surface conditions of high-performance bearings before and after the durability experiments.

After the testing of the high-performance bearings, the bearing surface will be worn to some extent. In order to more clearly analyze the bearing surface conditions, images of sample bearings were taken before and after the durability experiments. This can enable the naked-eye study and comparison of surface wear, as shown in Figure 6.

As shown in Figure 6, in the comparison of the bearings’ rolling elements, the surface glossiness of the tested rolling elements was obviously dim, and impurities penetrated into their surface. In the comparison of the cages, the surface of the tested cages had slight wear. There were slight indentations and burns on the parts that fit with the rolling body. In the comparison of the inner and outer rings, the surface gloss of the experimental inner rings of the bearings had become dim and had scratches. There were impurities infiltrated into the end face of the experimental outer ring, and there were obvious wear phenomena. The surface gloss of the inner and outer ring grooves was also dimmer than that of the inner- and outer-ring raceways of the bearings that were unserviced.

### 3.2. Metamorphic Layers

**Figure 7:**
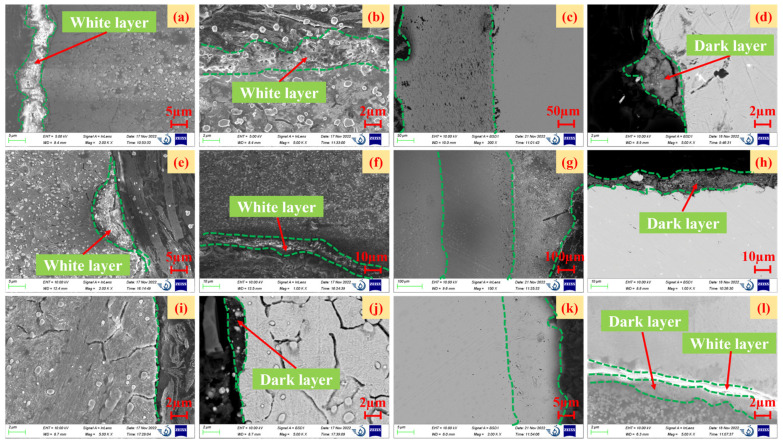
Comparison of the metamorphic layer of high-performance bearings before and after durability experiments.

According to the surface analysis in the previous section, the tested bearing surface had slight wear and impurity infiltration. This study also compared and analyzed the surface metamorphic layers of the inner- and outer-ring raceways and rolling bodies of the front-end and rear bearings, as shown in Figure 7. The formation of the metamorphic layer is mainly caused by the tempering of the matrix structure and the change in the grinding temperature. The metamorphic layer is divided into a “white layer” and a “dark layer”. The “white layer” has the characteristics of corrosion resistance, high hardness, fine grains, and high residual tensile stress, and microcracks with different angles generally exist in the “white layer”. Therefore, the “white layer” should be avoided in production practice. As for the “dark layer”, it is generally considered that it is caused by tempering of the matrix structure.

Figure 7a–d represent the metamorphic layers of the inner ring, outer ring, rolling body, and cage of the front-end serviced bearing, respectively. Figure 7e–h represent the metamorphic layers of the inner ring, outer ring, rolling body, and cage of the rear serviced bearing, respectively. Figure 7i–l represent the metamorphic layers of the inner ring, outer ring, rolling body, and cage of unserviced bearings, respectively.

According to the macroscopic analysis of Figure 7, in the experimental inner ring, outer ring, rolling body, and cage metamorphic layers of the front- and rear-end serviced bearings, there was not only a “white layer” (Figure 7a,b,e,f), but also a “dark layer” (Figure 7d,h). On the other hand, the inner ring (Figure 7i) of the unserviced bearing had almost no metamorphic layers, while the rest of the outer ring (Figure 7j), the rolling body (Figure 6k), and the cage (Figure 7l) also had metamorphic layers, and there were “white layers” and “dark layers” in the cage (Figure 7l).

The microscopic analysis Figure 7 shows that the maximum thickness of the metamorphic layers of the inner and outer rings of the front bearing was about 5 μm. The maximum “white layer” thickness in the cage (Figure 7d) was 6 μm. The maximum thickness of the metamorphic layers of the inner ring of the rear serviced bearing was 8 μm and 10 μm, respectively. In the cage (Figure 7h), the maximum “dark layer” thickness was 11 μm. However, there were essentially no metamorphic layers in the inner ring of the unserviced bearing. The maximum “dark layer” thickness of the outer ring (Figure 7j) was 3 μm. The maximum “white layer” and “dark layer” thickness in the cage was 1 μm and 2 μm, respectively.

### 3.3. Damage (Microscopic)

**Figure 8:**
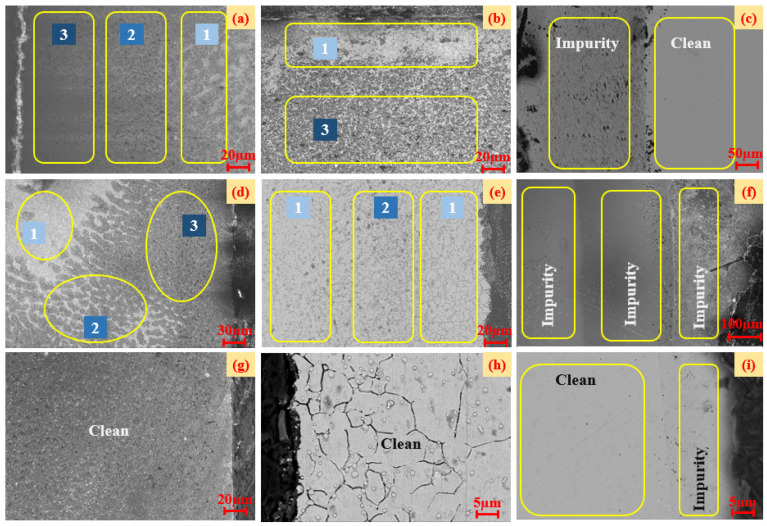
Comparison of the microscopic wear of high-performance bearings before and after durability experiments.

As shown in Figure 7, we also found two layers (Figure 7c,k) or even three layers (Figure 7g) of wear with different microscopic shapes on the surface of the rolling body from the metamorphic layers of the rolling body of the front-end, rear, and unserviced bearings after the experiment. In addition to the rolling elements, the inner ring of the front-end serviced bearing and the outer ring of the rear serviced bearing also had different levels of wear, as shown in Figure 8.

Figure 8a–c represent the microscopic wear of the inner ring, outer ring, and rolling body of the front-end bearing after the durability experiment, respectively. Figure 8d–f represent the microscopic wear of the rear bearing’s inner ring, outer ring, and rolling body after the durability experiment, respectively. Figure 8g–i represent the microscopic wear of the inner ring, outer ring, and rolling body of unserviced bearings, respectively. The numbers 1, 2, and 3 in Figure 8 represent the severity of different degrees of microscopic wear areas, where 1 is the lowest level of wear severity.

According to Figure 8, there were three different degrees of wear on the front-end bearing’s inner ring (Figure 8a), the rear bearing’s inner ring (Figure 8d), the rear bearing’s outer ring (Figure 8e), and the rear bearing’s rolling body (Figure 8f) after the experiment. After the experiment, the front-end bearing’s outer ring (Figure 8b), the front-end bearing’s rolling element (Figure 8c), and the unserviced bearing’s rolling element (Figure 8i) all had two different degrees of wear. The inner ring (Figure 8g) and the outer ring (Figure 8h) of the unserviced bearing did not have different degrees of microscopic wear.

### 3.4. EDS Analysis

**Figure 9:**
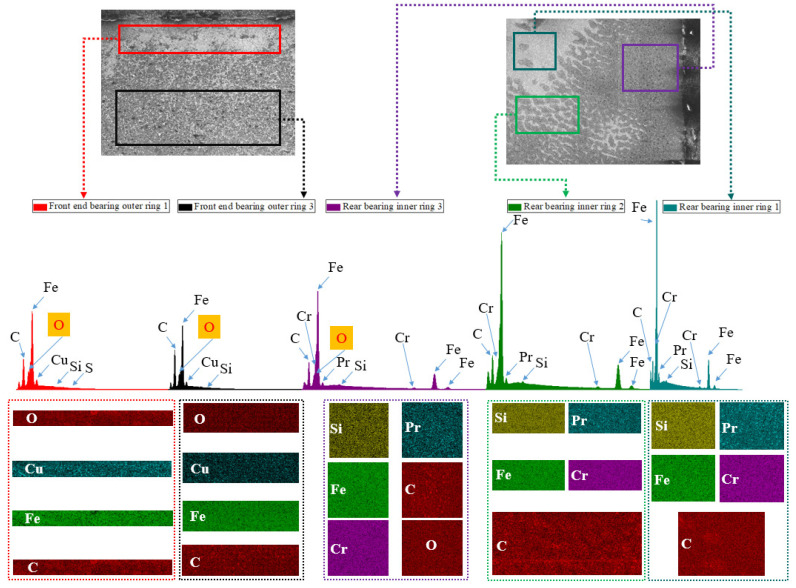
EDS of the wear delamination of the outer and inner rings of the front-end and rear high-performance bearings.

**Figure 10:**
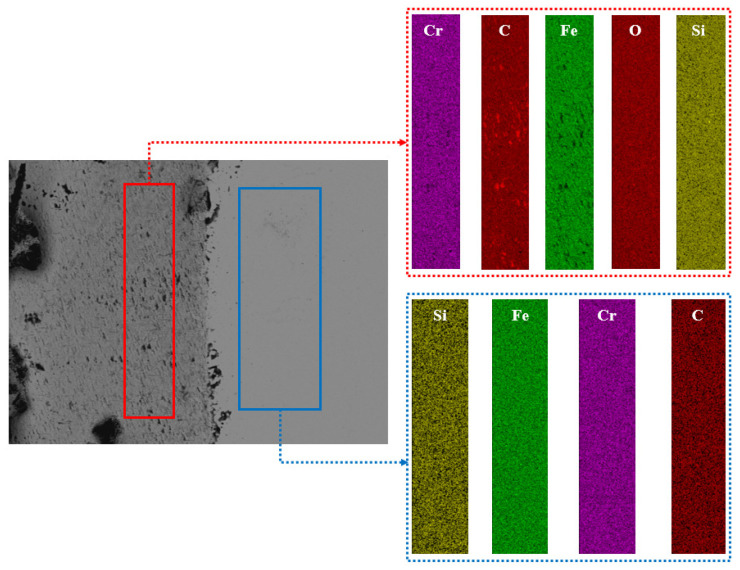
EDS of the front-end bearing’s rolling element wear delamination (serviced).

**Figure 11:**
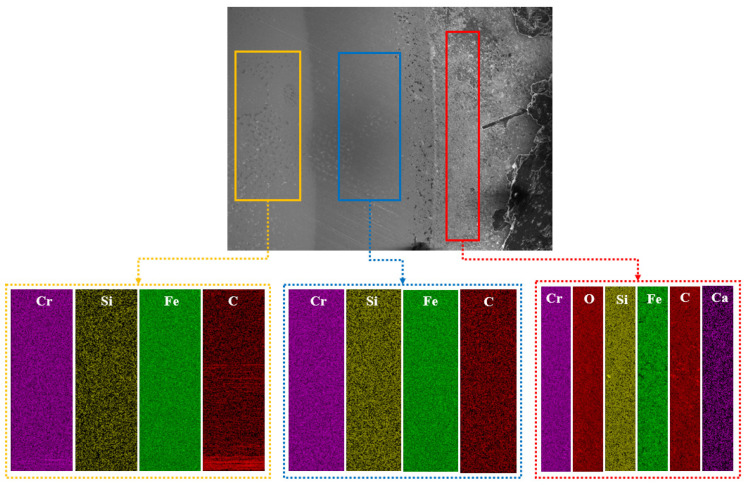
EDS of the rear bearing’s rolling element wear delamination (serviced).

**Figure 12:**
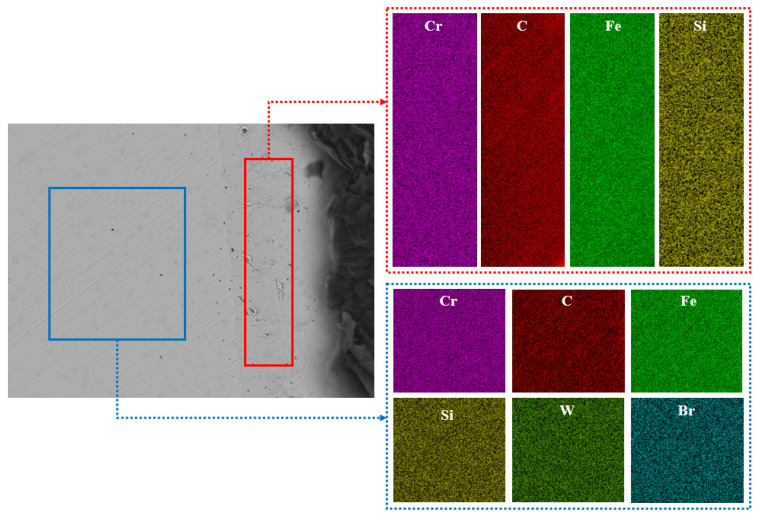
EDS of the wear delamination of bearings’ rolling elements (unserviced).

In this study, according to the different degrees of micro-wear in Figure 8, the layered EDS analysis of the inner and outer rings of the bearings after the durability experiments was carried out, as shown in Figure 9 and Table 1. Because the rolling elements were parts in direct contact with the inner- and outer-ring raceways, the rolling elements of the front-end and rear bearings after the durability experiment, as well as those of the unserviced bearings, were also analyzed by layered EDS, as shown in Figure 10, Figure 11 and Figure 12 and Table 2.

In Figure 9, front-end bearing outer ring 1 represents the area denoted by the number 1 in Figure 8b. Front-end bearing outer ring 3 represents the area denoted by the number 3 in Figure 8b. Rear bearing inner ring 3 represents the area denoted by the number 3 in Figure 8d. Rear bearing inner ring 2 represents the area denoted by the number 2 in Figure 8d. Rear bearing inner ring 1 represents the area denoted by the number 1 in Figure 8d.

The macroscopic analysis of Figure 9 and Table 1 shows that the front-end bearing’s outer rings contained the elements C, O, Fe, and Cu. The elements C, Fe, Si, Cr, and Pr were contained in the inner rings of the rear bearing. In addition, front-end bearing outer ring 1 also contained 0.3% elemental S. Rear bearing inner ring 3 contained 1.04% elemental O.

The microscopic analysis of Figure 9 and Table 1 shows that both front-end bearing outer ring 1 and front-end bearing outer ring 3 contained 0.24% elemental Si. The contents of elemental Fe and Cu in front-end bearing outer ring 1 were 4.16% and 0.98% more than those in front-end bearing outer ring 3, respectively. The contents of elemental C and O in front-end bearing outer ring 1 were 5.37% and 0.07% less than those of elemental Fe and Cu in front-end bearing outer ring 3, respectively. Meanwhile, among rear bearing inner rings 1, 2, and 3, rear bearing inner ring 3 also contained 1.04% elemental O. With the decrease in surface microscopic “spot” density shown in Figure 8d, the contents of elemental Fe and C decreased gradually, while the elemental Pr content increased gradually.

The macroscopic analysis of Figure 10, Figure 11 and Figure 12 and Table 2 shows that in the rolling element contents of the front bearing after the experiment, with the migration from the “ball edge” to the “ball center”, there was elemental C, O, Fe, Si, Ca, and Cr in the “ball edge”(the red part in Figure 10). In the “ball center”, which is the blue part in Figure 10, there was elemental C, Fe, Si, Cr, Al, and W. After the experiment, there were three layers of changes in the elemental contents of the rolling body of the rear-end bearing with the migration from the “ball edge” to the “ball center”. At the “edge of the ball”, which is the red part in Figure 11, there was elemental C, O, Fe, Si, Ca, Br, and Cr. The blue part in Figure 11 contained the elements C, Fe, Si, P, Cr, Al, and Sr. In the “ball center”, which is the yellow part in Figure 11, there was elemental C, Fe, Si, and Cr. In the rolling element contents of the unserviced bearings, with the migration from the “ball edge” to the “ball center”, the elements C, Fe, Si, Ca, and Cr were contained in the “ball edge”, which is the red part in Figure 12. In the “ball center”, which is the blue part in Figure 12, there was elemental C, Fe, Si, Br, Cr, and W. Meanwhile, the biggest difference between the unserviced and serviced rolling bodies was whether they contained elemental O.

The microscopic analysis of Figure 10, Figure 11 and Figure 12 and Table 2 showed that the front-end and rear bearing rollers after the experiments contained 6.23% and 4.63% elemental O, respectively, while the unserviced bearing rollers did not contain elemental O. The contents of C and Fe in the “ball edge” were always smaller than those in the “ball center”, regardless of whether the rolling elements of the bearings were serviced or not, and C and Fe were the most varied elements in the contents of each layer (color). As shown in Figure 10, the difference in the contents of C and Fe between the “sphere edge” and the “ball center” was 7.22% and 13.68%, respectively. As shown in Figure 11, the difference in the contents of C and Fe between the “ball edge” and the “ball center” was 5.43% and 8.5%, respectively. As shown in Figure 12, the difference in the contents of C and Fe between the “ball edge” and the “ball center” was 1.91% and 5.39%, respectively. Meanwhile, it was found that the contents of C in the “ball edge” and “ball center” decreased slowly, while the contents of Fe increased slowly.

## 4. Comprehensive Analysis

Based on the comprehensive analysis of Section 2 and Section 3 of this paper, the following can be derived:

(1) In Section 2 of this study, the total durability test time was 1508 h, including the high-speed (8 h), high-temperature (300 h) and heavy-load (1200 h) experiments. The bearing life was calculated according to Equation (1) [34,35]:

(1) $$L_{10}={\left(\frac{C}{P}\right)}^{3}\times \frac{10^{6}}{60n}$$

where *C* is the basic rated dynamic load (122 kN), *P* is the equivalent dynamic load (17.07 kN), and *n* is the average speed (4108 r/min); these values were derived from the actual working conditions.

The calculated life of the bearing was 1481 h, which was less than the running life of the bearing (1508 h). The operating temperature was higher than the normal temperature, leading to a decline in the actual life of the bearings. Therefore, the rated dynamic load of the bearing was greater than 122 kN.

**Figure 13:**
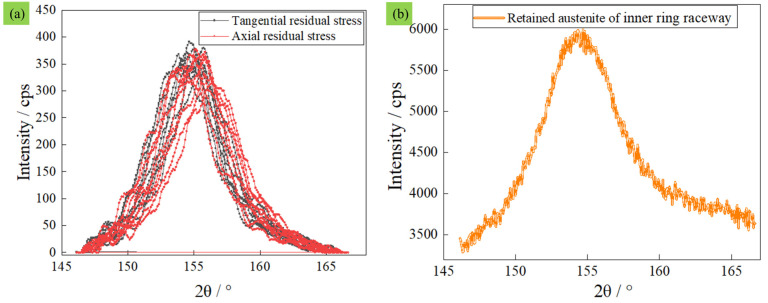
Scanning peak intensity of residual stress and retained austenite in a bearing’s inner-ring raceway. (**a**) Scanning peak intensity of residual stress. (**b**) Scanning peak intensity of retained austenite.

(2) In Section 3 of this paper, the metamorphic layers of the high-performance bearings (Figure 7) were analyzed, and the following sequence was determined: the metamorphic layers of rear bearing (i.e., inner ring, outer ring, rolling body, and cage) > the metamorphic layers of the front-end bearing > the metamorphic layers of the unserviced bearing. Combining the results shown in Figure 9 and Table 1, the contents of C and Fe in the inner ring of the rear bearing were less than those in the outer ring of the front-end bearing, while the contents of Cr and Pr were greater. Compared with the EDS analysis of the unserviced rolling body (containing the elements C, Fe, Si, Ca, Br, Cr, and W), the rolling body after the experiment also contained elemental O, P, and Sr. However, the presence of O in the bearings will accelerate their internal oxidation and lead to a decrease in their fatigue life. The content of elemental P in the bearing steel was generally no more than 0.030–0.040%, but the presence of 0.06% elemental P will cause serious segregation, increase the tempering brittleness, significantly reduce the plasticity and toughness of the bearing steel, and cause it to be prone to brittle cracking during cold working. At high temperatures, elemental Sr can easily react with water and acid to release hydrogen, which will damage the fatigue strength of the bearings. Therefore, in the use of bearings, the lubricating medium and sealing mode of the bearings are the key measures to improve the fatigue life of the bearings. On the other hand, with the migration from the “ball edge” to the “ball center”, the adsorption capacity and contents of elemental C will decrease gradually, leading to a decrease in the surface hardness of the rolling body and, ultimately, to fatigue failure of the rolling body.

(3) Residual stress and retained austenite are two of the important indices to evaluate the surface integrity of bearing grooves [36,37]. Because the metamorphic layers of the rear bearing were larger than the metamorphic layers of the front-end bearing, the residual stress and retained austenite of the inner-ring and outer-ring raceways of the rear bearing were measured. The tangential residual stress, axial residual stress, and retained austenite in the inner-ring raceway were −991.8 MPa, −1353.5 MPa, and 11.4%, respectively. The tangential residual stress (Figure 13a), axial residual stress (Figure 13a), and retained austenite (Figure 13b) in the outer-ring raceway were −942.1 MPa, −1043.1 MPa, and 13.7%, respectively. These values easily meet the requirements of actual working conditions. Of course, if we want to make these characteristics better, we need to analyze the bearings’ grinding process.

## 5. Conclusions

In this study, the wear and metamorphic layers of the front-end and rear bearings were observed by analyzing the high-speed (lasting 8 h), high-temperature (lasting 300 h), and heavy-load (lasting 1200 h) durability experiments of high-performance bearings. In view of the wear and metamorphic layers, a multidimensional comprehensive comparison between the serviced and unserviced bearings was carried out, and the conclusions were as follows:

(1) In the high-speed, high-temperature, and heavy-load durability experiments, the experimental temperature of the front-end bearings was always lower than that of the rear bearings. In the high-speed endurance experiment, when the speed was stable at a given time, the temperature of both the front-end and rear bearings increased sharply. In the high-temperature durability experiment, with the increase in speed (minimum speed 3000 r/min, maximum speed 6300 r/min) and time, the maximum temperature of the front and rear bearings was 36.7 °C and 85.8 °C, respectively. In the heavy-load durability experiment, with a constant speed (3000 r/min) and the increase in time, the maximum axial loads of the front-end and rear bearings were 23,076 N and 583 N, respectively.

(2) After the experiments, the surface glossiness of the front-end and rear bearings decreased obviously (including the inner ring, outer ring, rolling body, and cage). The metamorphic layers of the serviced rear bearing (i.e., inner ring, outer ring, rolling body, and cage) > the metamorphic layers of the serviced front-end bearing > the metamorphic layers of the unserviced bearing. In the EDS analysis of the bearings after the experiments, the outer ring contained elemental C, O, Fe, Si, S, and Cu. The inner ring contained elemental C, O, Fe, Si, Cr, and Pr. With the migration from the “ball edge” to the “ball center”, the C content of the rolling body of serviced or unserviced bearings decreased slowly, while the content of Fe increased slowly. However, in actual working conditions, the infiltration of some harmful elements is inevitable. In order to reasonably control the occurrence of surface-layer deterioration, it is necessary to study the processing of bearings’ raw materials and precision grinding technology.

(3) Comprehensive analysis showed that the thickness of the rear bearing at high temperatures in the high-speed, high-temperature, and heavy-load durability experiments was greater than that of the front-end bearing at low experimental temperatures. Similarly, the number of metamorphic layers of the rear bearings (including the inner ring, outer ring and rolling body) at high test temperatures was greater than the number of metamorphic layers at low test temperatures. Moreover, the rolling body of the rear bearing at high experimental temperatures contained not only elemental O, but also elemental P and Sr. These three elements have a great impact on the surface integrity of the rolling body when in service. Therefore, the surface of the rolling body is damaged, resulting in impurities being adsorbed on the surface of the rolling body. This further affects the surface integrity of the inner- and outer-ring raceways of the bearings and reduces the effective service cycle of high-performance bearings. In subsequent work, we will analyze the thickness of the metamorphic layer throughout the precision grinding process of bearings. In the service of bearings, the grinding process can be optimized by analyzing the thickness of the metamorphic layer by EDS, so as to prolong the service life of the bearings.

(4) A very important problem has been solved by the experimental investigation of high-performance bearings for up to 1500 h. Before the bearing is used, it is necessary to predict the actual working conditions. It is very necessary to deal with the processing technology of front and rear bearings separately. That is, the front- and rear-end bearings cannot be randomly used under a batch production process. This problem suggests that many manufacturers and users need to select bearings with different performances as front and rear bearings to ensure the stability of weapons and equipment and prolong their service life. In the analysis of the metamorphic layer, elemental contents, residual stress, and retained austenite, it is suggested that bearing manufacturers should control the grinding temperature and grinding force during precision grinding or lapping of the bearings’ raceways. The grinding temperature and grinding force will directly affect the surface fatigue strength of high-performance bearings, thereby affecting their service life. Therefore, in subsequent work, we will analyze the thickness of the metamorphic layer throughout the precision grinding process of bearings. The retained austenite and hardness of the surface and inner layers of high-performance bearing raceways will be analyzed. In the process of wear, grain refinement will occur in the surface layer of the raceway. Based on the grain refinement model of high-performance bearings and the thickness of the modified layer analyzed by EDS, the grinding process can be optimized to prolong the service life of the bearings.

## Figures and Tables

**Table 1:** EDS results of the wear delamination of the inner and outer rings of the front-end and rear bearings.

Project	C (wt%)	O (wt%)	Fe (wt%)	Si (wt%)	S (wt%)	Cu (wt%)	Cr (wt%)	Pr (wt%)
Front-end bearing outer ring 1	9.99	3.03	81.32	0.24	0.30	5.12		
Front-end bearing outer ring 3	15.36	3.10	77.16	0.24		4.14		
Rear bearing inner ring 3	8.82	1.04	57.56	0.17			2.68	29.73
Rear bearing inner ring 2	6.25		56.84	0.18			6.68	30.04
Rear bearing inner ring 1	3.97		52.73	0.17			5.18	37.94

**Table 2:** EDS of the wear delamination of high-performance bearings’ rolling bodies before and after the durability experiments.

Figure	Color	C (wt%)	O (wt%)	Fe (wt%)	Si (wt%)	Ca (wt%)	P (wt%)	Br (wt%)	Cr (wt%)	Al (wt%)	W (wt%)	Sr (wt%)
Figure 9	Red	10.98	6.23	77.03	0.72	0.24			4.79			
	Blue	3.76		90.71	0.21				5.11	0.05	0.16	
Figure 10	Red	9.23	4.63	81.59	0.50	0.21		0.35	0.61			
	Blue	3.6		91.00	0.29		0.06		4.79	0.08		0.19
	Yellow	3.8		90.09	0.20				5.91			
Figure 11	Red	6.78		84.54	0.26	0.07			4.62	0.1		
	Blue	4.87		89.93	0.22			0.21	4.62		0.14	

## Data Availability

The data presented in this study are available on request from the corresponding author.
